# A Practical Guide to the Utilization of ChatGPT in the Emergency Department: A Systematic Review of Current Applications, Future Directions, and Limitations

**DOI:** 10.7759/cureus.81802

**Published:** 2025-04-06

**Authors:** Nathaniel S Meyer, John W Meyer

**Affiliations:** 1 General Practice, Eisenhower Army Medical Center, Fort Eisenhower, USA; 2 Emergency Medicine, Penn State College of Medicine, Hershey, USA

**Keywords:** artificial intelligence, chatgpt, differential diagnosis, digital health communications, ekg, large language model, medical decision-making, medical documentation, triage, workflow

## Abstract

The rapid development of artificial intelligence (AI) tools across various medical specialties highlights the potential for AI to transform medicine over the next 20 years. Despite this potential, the adoption of AI can feel incremental and disconnected from the daily practice of individual clinicians. For emergency department (ED) physicians practicing in 2025, recognizing and evaluating AI tools available for immediate integration into practice is essential. One such tool is ChatGPT (OpenAI, San Francisco, California, United States), a large language model (LLM) that is free, easily accessible via smartphones or computers, and widely used across industries. However, its usability in the ED setting remains poorly characterized. This review explores the current evidence surrounding ChatGPT 4's applications in various ED physician tasks, documenting its strengths and limitations. While ChatGPT demonstrates significant utility in language generation and administrative tasks, its potential for supporting more complex tasks in medical decision-making is emerging but not yet robust. The available evidence is limited and variable and lacks standardization, reflecting a field still in its early stages of development. Notably, the performance improvements observed between ChatGPT 3.5 and ChatGPT 4 suggest that future iterations, such as the anticipated release of ChatGPT 5, could significantly impact these findings. This review provides a comprehensive snapshot of the current state of evidence regarding ChatGPT's use in the ED, offering both an evaluation of its capabilities and a practical guide for its appropriate use by ED clinicians today.

## Introduction and background

Since its initial release to the general public in November 2022, OpenAI's ChatGPT large language model (LLM) (San Francisco, California, United States) has taken the world by storm. Benefiting from extensive media coverage and rapid infiltration into pop culture, it became a ubiquitous tool almost overnight. By March 2023, version 4.0 was released, fulfilling promises of increased fidelity and further solidifying its role as a transformative technology. This freely available tool has changed the way people read, write, and search for information. Across industries, it has sparked both hope and concern, with medicine being no exception. From firsthand accounts, physicians have already begun exploring ChatGPT to evaluate its potential for integration into clinical practice. However, the frequent updates to the model have posed challenges to researchers, causing published studies to lag behind and quickly risk obsolescence.

While artificial intelligence (AI) is widely researched across many disciplines of medicine, most applications undergoing development rely on machine learning algorithms integrated into specific tools or electronic medical records (EMRs). These purpose-built tools are implemented at a systems-based level, where individual clinical physicians have limited control over their use. In contrast, ChatGPT is free, portable, and available for immediate use, making it uniquely poised to impact the day-to-day clinical practice of individual physicians. Although ChatGPT was not originally designed for medical applications, its accessibility justifies exploration into how this technology might complement physician efforts.

ChatGPT has garnered significant attention for its potential to streamline administrative tasks, enhance communication, and support clinical decision-making. Emergency departments (EDs), characterized by high patient volumes, diverse case presentations, and time-sensitive demands, represent a particularly compelling environment for such tools. ChatGPT's potential applications in the ED include aiding with documentation, providing rapid access to medical literature, and generating patient education materials.

By its nature as an LLM, ChatGPT utilizes complex statistical calculations and vast amounts of human text to generate coherent and contextually relevant responses. While it does not possess true understanding or general intelligence, its outputs can often achieve a level of fidelity that approximates such intelligence.

Emergency medicine, a specialty grappling with increasing rates of physician burnout yet recognized for its ingenuity, may stand to benefit from novel solutions like ChatGPT. The aim of this review is to capture the currently available research pertaining to ChatGPT's potential applications for emergency medicine physicians. Based on the limited data currently available, we attempt to offer evidence-based guidance on what applications may benefit current practice and what applications may be of benefit to clinical practice in the near future and offer precautions against practices with significant risk. The use of ChatGPT in both language generation tasks and medical decision-making tasks, as well as its inherent limitations, will be addressed.

## Review

Methods

This systematic review was conducted to evaluate the applications of ChatGPT in emergency medicine. Searches were performed using Google Scholar due to its wide variety of academic sources. The following Boolean search strategy was used: ("ChatGPT" OR "Chat GPT" OR OpenAI) AND ("Emergency Medicine" OR "Emergency Department"). The last time this search term was executed for data gathering was on December 11, 2024. The search terms were intended to cast a broad net including any papers that mentioned both ChatGPT and a term relevant to emergency medicine. The search was limited to English-language articles published from 2022 to 2024, reflecting the public release of ChatGPT in November 2022. An initial total of 4,550 results were retrieved.

Screening and Eligibility

Screening was conducted in two phases.

Primary screening: Initial search was conducted using the Boolean search phrase above in Google Scholar. This initial search yielded 4,550 results. Duplicates of potentially relevant articles were manually excluded totaling 26 articles, and duplicates of obviously irrelevant articles were not assessed due to their exclusion in phase 1 of screening. Titles and keyword snippets were screened manually by two reviewers independently for relevance based on predefined inclusion and exclusion criteria. In the event of disagreement between the two reviewers, individual articles were discussed, and inclusion/exclusion required unanimous agreement. Inclusion criteria required mention of at least one item from each of the following two categories: (1) artificial intelligence (e.g., AI, ChatGPT, OpenAI, large language model, LLM) and (2) emergency medicine (e.g., emergency department, trauma, triage, imaging, EKG). Exclusion criteria included not meeting the inclusion criteria or being articles that were focused on a different, specific specialty (e.g., ophthalmology, public health), addressed non-physician use cases (e.g., nursing, prehospital care, healthcare administration), discussed only versions of ChatGPT predating 4.0, examined AI programs unrelated to ChatGPT, or centered on the use of ChatGPT specifically for board exam preparation. A total of 125 potentially relevant articles were identified. The high attrition rate from 4,550 to 125 was largely attributed to publications including brief mentions of ChatGPT, for instance, crediting ChatGPT with contributing to writing or editing assistance, yet not substantively discussing its role in emergency medicine. 

Secondary screening: All 125 article abstracts were then reviewed manually. Inclusion criteria required that the study demonstrate the use of ChatGPT 4 to perform a task relevant to emergency medicine physicians. Articles that did not meet the above relevance criteria or that reintroduced any previously stated exclusion criteria for phase 1 were discarded. Following this secondary screening, 46 articles were included for analysis and synthesis, although the annotation phase described below revealed that nine of these 46 articles did not possess original findings or substantive commentary.

Data Extraction and Analysis

**Figure 1:**
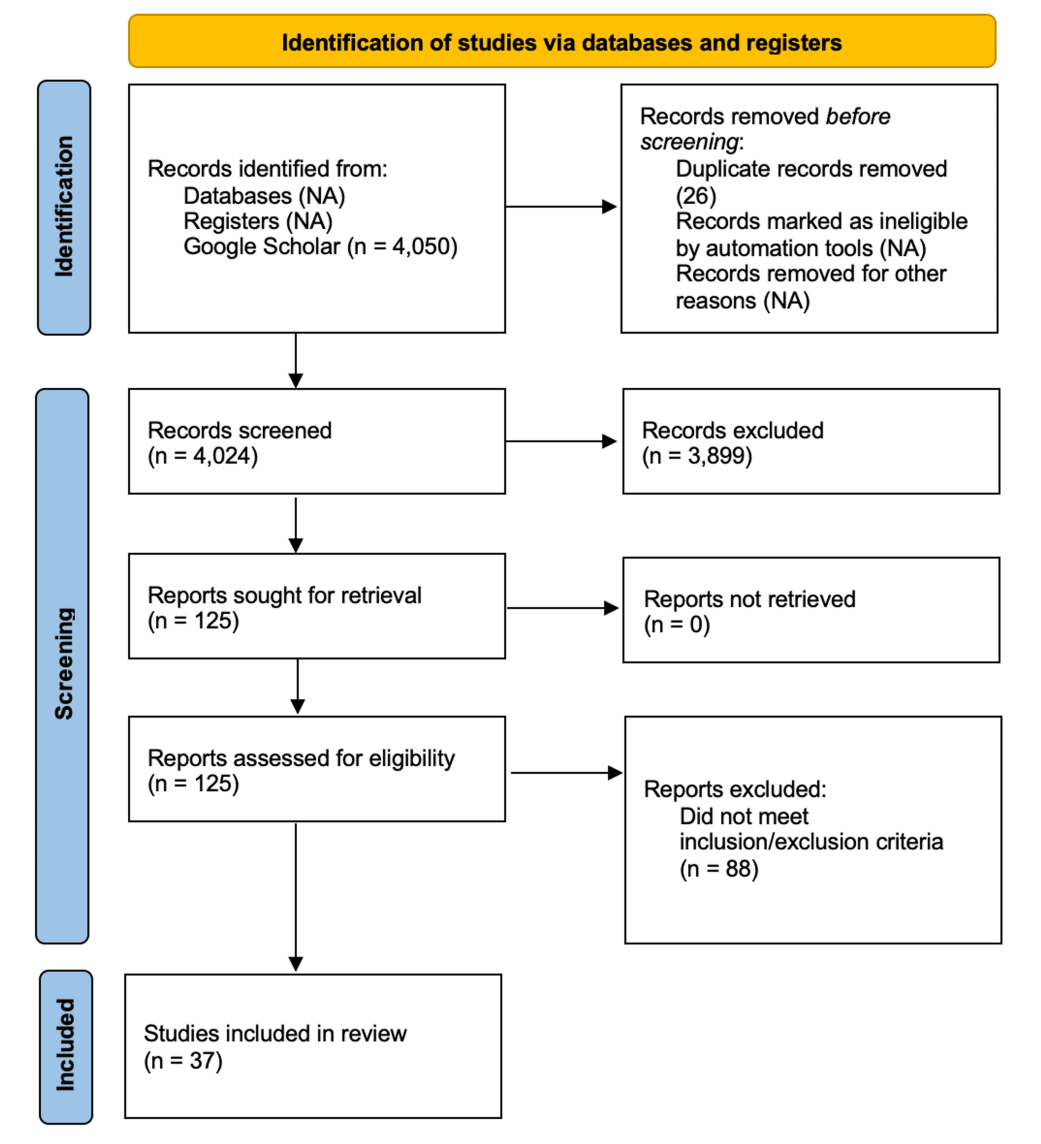
PRISMA systematic review method flowsheet PRISMA: Preferred Reporting Items for Systematic Reviews and Meta-Analyses

Full-text versions of these 46 articles were stored using the Zotero research assistant program (Corporation for Digital Scholarship, Vienna, Virginia, United States). Each reviewer manually annotated articles for content and tagged manuscripts based on relevant topics. Trends, levels of evidence, and themes regarding ChatGPT's utility in emergency medicine were extracted. Content tags were used to organize sources into body sections. Findings from these studies guided the structure of this review, synthesizing information to highlight current evidence, potential applications, and limitations of ChatGPT in emergency medicine. After thorough analysis and annotation, it was determined that nine articles meeting our inclusion criteria did not offer substantive new findings. These articles were opinion pieces or letters to editors of various journals and did not contain or cite relevant research, and they were not included in our final manuscript. Potential bias was mitigated by utilizing independent but concurrent review, annotation, and synthesis. Disagreements were discussed, and ultimately, both sides of every argument are presented in this manuscript with level-of-evidence notation as appropriate. Per the Preferred Reporting Items for Systematic Reviews and Meta-Analyses (PRISMA) guidelines for systematic review data collection methods, a flowsheet is available below in Figure 1 outlining the process.

Language generation applications

Documentation

Among the various potential uses for ChatGPT in emergency medicine, its core strength, that is, text generation, positions it as a promising resource for mitigating the documentation burden on physicians. Administrative tasks and charting are frequently cited as key contributors to emergency physician burnout, and integrating AI-driven solutions has been proposed as a way to ease this cognitive load and allow for more time and energy to be devoted to direct patient care [1].

Before discussing ChatGPT's specific capabilities, it is important to acknowledge existing AI-driven scribe tools designed explicitly for clinical documentation, such as Suki AI (Redwood City, California, United States), Augmedix (San Francisco, California, United States), Scribekick AI (Richmond, Virginia, United States), and Notable Health (San Mateo, California, United States). These programs often incorporate specialized audio equipment to capture and summarize patient encounters, thereby functioning as "AI scribes." While they can be highly effective, they are typically expensive and require deployment at the hospital or health system level, an implementation process over which individual emergency medicine physicians generally have little control. By contrast, ChatGPT is freely available and accessible from any internet-connected device, making it an appealing option, even though it is not optimized for charting.

Recent pilot studies have examined ChatGPT's utility in generating clinical documentation, including attending attestation notes [2], discharge summaries [3], and I-PASS handoffs [2]. These studies involved prompting ChatGPT with non-identifying patient data extracted from the EMR. While the AI-generated text was generally accurate and sometimes saved time, emergency physicians felt the need for careful review was subjectively draining, and it often negated time savings [2].

In one study focused on ChatGPT-generated discharge summaries, only 33% of the AI-generated summaries were initially error-free [3]. However, in post-hoc analysis, it was found that prompt design played a large factor. By modifying input prompts minimally, they increased the proportion of error-free summaries to 47% [3]. The findings are promising, as input of non-identifying patient information can almost instantly generate a passable note in many cases, with minimal edits required in other cases. These findings also suggest that prompt design plays a crucial role in the accuracy of ChatGPT's outputs. They also demonstrate a limitation of ChatGPT, namely, the requirement for close physician oversight to correct subtle or critical errors that may occur in any section of the text. Ultimately, these data suggest that the utility of ChatGPT to aid documentation will hinge upon physician preference; if the time spent writing notes outweighs the time spent analyzing and editing AI-generated notes, then AI-generated notes could expedite the documentation process. However, these two studies are small pilot studies that generally failed to generate statistically significant results. Larger and more controlled studies are needed before definitive conclusions can be drawn.

Additionally, no studies were found commenting on the use of ChatGPT to generate templates or smart phrases. This application may be more naturally suited for ChatGPT than the generation of entire encounter notes. To sample its capabilities, the following prompts were given to ChatGPT, and output examples are included in Appendix A: "generate a procedure template for laceration repair" and "generate a template for medical decision-making for an emergency room encounter". With minor edits, these templates could be saved to a user profile in an EMR. The barrier to the generation of templates is appreciably lowered using this strategy, so a user could build a vast library of pre-made templates of various types of encounters with relative ease.

Communications

ChatGPT also shows promise in facilitating communication, both among healthcare providers and between physicians and patients. In many industries, it is already routinely used for composing emails and handling personal assistant-type tasks. Specific to emergency medicine, limited evidence exists specifically addressing the use of ChatGPT in improving communications between emergency medicine physicians and patients. Readers of this article are encouraged to attempt sample texts themselves to determine if this application would benefit their workflow, keeping in mind that the writer maintains full responsibility for any communications sent. It is unlikely that this application will have evidence published in a journal concerning it, as it is more of an intuitive use case.

Patient Instructions and Discharge Materials

When discharged from the ED, instructions for ongoing care, follow-up, and return precautions can sometimes be lost in translation due to complex terminology or medical jargon. One study used simulated ED scenarios to generate ChatGPT-based discharge instructions and then enlisted members of the general public to compare them against physician-written instructions [4]. While the study was small and did not reach statistical significance in most outcomes, participants reported higher levels of interpretability, understanding, and satisfaction with the AI-generated materials. Notably, ChatGPT significantly outperformed physicians (p=0.01) in the interpretability of return precautions section, suggesting the model's unique ability to simplify complex information without losing critical details [4]. Again, these discharge instructions would require close oversight, but ChatGPT's capacity to instantly modify tone and readability shows promise for bridging communication gaps. Even more promising would be the use of ChatGPT to generate discharge instruction templates, edit templates for accuracy, and then have templates available for subsequent similar visits. A similar study that examined the use of ChatGPT-generated discharge letters for pediatric ED encounters found that AI-generated instructions were both time-saving and appropriately tailored to patient reading levels [2]. Additionally, these letters were found to be generally accurate [2].

Addressing Patient Concerns

Although answering patient concerns is not generally addressed directly by emergency medicine physicians, certain situations demand direct physician involvement. In one study, ChatGPT-generated responses to hospital complaints were rated more favorably than human-authored responses, with reviewers citing greater clarity and empathy [5]. Interestingly, the AI answers were rated higher in empathy, possibly due to the machine's unfeeling nature and inability to take offense at complaints. This somewhat counterintuitive finding may stem from the AI's neutral tone and its inability to react defensively, suggesting a potential role for ChatGPT in crafting or streamlining sensitive communication.

Visual Communication and DALL-E Integration

With the integration of DALL-E (OpenAI, San Francisco, California, United States), an AI image processor and image generator, into ChatGPT in October 2023, AI-generated images add an entirely new facet to ChatGPT's potential applications. Some researchers have studied these image generation capabilities to bridge gaps in communication with children, who obtain less benefit from written instruction. ChatGPT was used to generate bright, colorful, calming images of minor procedures before children had to undergo procedures themselves. In this proof-of-concept study, researchers observed that children who viewed AI-generated images that demonstrated what their experience would entail seemed to be less anxious and more cooperative and retained health information better than those who did not [6]. In Appendix B, three images were generated using similar methodology to provide the reader a sample of current capabilities. Beyond pediatric use, these image generation features may have broader applications, such as bridging language or cultural barriers and assisting individuals with intellectual disabilities. However, readers should be cautioned about the use of AI image generation. Its current iteration may prove unpredictable and more challenging to moderate than text-based outputs. Point-of-care use may result in the inadvertent generation of an inappropriate image that may seem appropriate upon initial viewing, an especially concerning risk when working with vulnerable populations. For example, in this study, when ChatGPT was tasked to generate an image of a three-year-old girl receiving an abdominal ultrasound, the generated image appears to have depicted a fetus on the ultrasound monitor, which is both bizarre and potentially offensive.

Medical decision-making

ChatGPT's nature as an LLM makes its ideal use case in processing and generating language. It relies on a vast array of linguistic data to make optimal statistical predictions from word to word and phrase to phrase to generate the most probably accurate text. Although it lacks genuine understanding and has only a limited capacity for logical reasoning, it can be fine-tuned and instructed to perform tasks that increasingly approximate true clinical logic. With this qualifier, use cases under study for ChatGPT's relevance to ED decision-making will be discussed below.

Triage

ChatGPT ED triage is the most widely researched topic in our review of current literature. Ten studies were reviewed that investigated ChatGPT's ability to triage patients, and one meta-analysis was compiled. Although the findings vary significantly across these sources, a few overarching patterns are apparent.

Of the 10 experimental studies, six reported that ChatGPT's accuracy either matched the level of a resident or approached the gold standard [7-12]. The remaining four experiments noted serious deficiencies in overall accuracy, generally trending towards moderate levels of agreement with the gold-standard triage procedure [13-16]. A 2024 meta-analysis found that ChatGPT demonstrated a pooled accuracy regarding correctly assigning triage status of 86% [17]. However, in agreement with our analysis, these authors were concerned with high variability and potentially biased distribution [17]. Several studies were conducted using variously sourced clinical vignettes; some used simulated cases found in triage reference materials, and others used real, anonymized patient data. Even between groups that studied anonymized patient data, significant variability between anonymization procedures exists. For example, some studies only input symptoms and vital signs, while others gave ChatGPT access to a patient's full anonymized medical history and chart data. Significant variation also existed within the prompt engineering of these studies. Additionally, performance variability between simulated reference materials and real patient encounters is not certain.

Notably, one study accomplished especially compelling results through developing a novel methodology. Rather than using an untrained version of ChatGPT, where it has not been used previously and learned user preferences, the research team specifically trained the model on a particular triage metric and institutional modifications to triage zones. By supplying a large amount of anonymized EMR data, ChatGPT achieved near-perfect agreement with gold-standard triage procedures [11]. While the other studies above struggled with small samples and vignette-based problem-solving, this study was both well-powered and prospective.

An interesting pattern observed is ChatGPT's tendency to over-triage patients to higher acuity levels than warranted [9,10,16]. It is unclear if this trend is intrinsic to LLMs or if the LLM is intentionally programmed to err on the side of caution. Potentially as a result of the over-triage tendency, it has been found repeatedly that ChatGPT performs best at the highest level of acuity [12,13,15]. One possibility is that ChatGPT's recognition of severe symptoms is especially robust, while another possibility is that once the highest acuity tier is reached, there is no room left for excessive escalation.

Another trend observed between studies of triage data is that ChatGPT 4 performs significantly better than ChatGPT 3.5 [7,17]. While studies exclusively containing older iterations of ChatGPT were not included in this review, multiple studies included side-by-side comparisons of versions 3.5 and 4, consistently demonstrating better accuracy with the latter. It remains uncertain whether future iterations will sustain this trajectory of advancement or eventually plateau. Regardless, with the release of ChatGPT 5 on the horizon, repeated studies will be critical for validating the model's progress.

Differential Diagnosis

Anecdotal accounts of ChatGPT's use in generating a differential diagnosis have circulated in hospitals since its initial public release. In an effort to quantify the model's performance, multiple research groups have conducted preliminary evaluations. Overall findings appear to be less variable than those observed in triage applications; however, conclusions remain difficult to draw due to limited evidence and non-standardized methodologies. When ChatGPT was tasked to generate differential diagnoses from a reference case with pre-established differentials, ChatGPT was found to list only 63% of appropriate diagnoses [18]. However, this same study found that ChatGPT listed several plausible diagnoses not listed in the reference text. Other studies tested ChatGPT's ability to generate differentials versus physicians using data from real patient encounters. One experiment showed that ChatGPT significantly outperformed residents in differential accuracy, and another found no statistical difference between ChatGPT and attending physicians [19,20]. A meta-analysis likewise showed ChatGPT outperforming other similar LLMs, performing at about the same level as a non-expert physician [21]. In other words, ChatGPT performed as well as a neurosurgeon in generating diagnoses in an obstetrics case but was clearly outperformed by a neurosurgeon's analysis of a neurosurgical case. These findings are further supported by Hoppe et al. [20], which showed that ChatGPT performed at or above the level of an emergency medicine resident in establishing a single diagnosis.

With the limited evidence available, it would appear that the ability of a physician to generate differential diagnoses using ChatGPT is both robust and somewhat unpredictable. Its most beneficial application in current practice may be to "rule in" additional considerations rather than definitively "rule out" any potential diagnoses. For example, a physician would first generate a differential in his/her typical manner. Based on the findings by Altamimi et al. [18], ChatGPT could generate diagnoses that align with most entries on the physician's list while also suggesting an additional plausible condition the clinician had not yet considered. The physician would then quickly determine which diagnoses are erroneous and determine if ChatGPT has provided a helpful diagnosis that requires further workup. Such usage is both reasonable and exciting, provided the physician does not exclude valid diagnoses missed by ChatGPT or succumb to confirmation bias in favor of the model's recommendations. In this capacity, ChatGPT can serve as a supportive tool in decision-making, contributing an extra layer of insight rather than acting as a stand-alone diagnostic authority.

Electrocardiogram (EKG)

As discussed previously in Communications, the integration of DALL-E into ChatGPT adds powerful image encoding and decoding capabilities. However, image analysis and generation have been noted to be less predictable than language functions. Minimal evidence currently exists surrounding the ability of ChatGPT to interpret EKGs. Two studies have compared ChatGPT's ability to solve textbook multiple-choice EKG questions, comparing its performance with emergency medicine and cardiology physicians. In one of these studies, ChatGPT outperformed both emergency medicine and cardiology physicians and tended to be most accurate in routine EKGs compared to more challenging EKGs [22]. The other study found that ChatGPT was inferior to both emergency medicine and cardiology physicians and tended to perform best on more challenging EKGs compared to routine [23]. Notably, these contradictory findings came from the same research group using the same textbook for EKG analysis. Additionally, no published data exist on ChatGPT's use in real patient EKG interpretation, where artifacts may pose a challenge. As a result, it is not advisable to interpret EKGs using the program in its current iteration, as the anchoring bias from a false answer likely outweighs any potential benefit from this mostly untested methodology. Perhaps a more interesting direction for future research would be to assess ChatGPT's EKG analysis against the computer-generated EKG interpretation already found on most EKG strips, an approach aligned with ChatGPT's intended purpose as a supplementary tool rather than a replacement for physician judgment.

Decision Support

ChatGPT's role as a potential decision support tool has recently begun to garner attention. Compared to decision support systems being developed using machine learning for specific reasoning tasks, there is concern that ChatGPT can exhibit unpredictable behavior given its lack of robust, intrinsic logical capabilities. Instead of directly performing logic functions to determine likelihoods, it approximates logic by drawing on patterns derived from outside linguistic data. Despite these limitations, ChatGPT's free availability and easy accessibility make it an intriguing option, and it should not be altogether dismissed.

While evidence is limited across various decision support applications, radiologic topics have the most published evidence. Two small pilot studies used input from real patient encounters to prompt ChatGPT to determine whether ChatGPT could make evidence-based recommendations on what radiologic studies are clinically indicated. In one study, ChatGPT achieved 40 out of 40 correct suggestions based on the American College of Radiology Appropriateness Criteria, while another study reported 85 out of 94 correct recommendations using the European Society of Radiology iGuide [24,25]. The latter group noted that accuracy could further improve if physicians reviewed ChatGPT's outputs in practice. Several of the few incorrect answers were nonsense answers. For example, a recommendation for interstitial lung disease workup was provided in an abdominal pain presentation [25]. Under the assumption that a physician would discard such outliers, the overall usefulness of the tool would increase. However, another larger study showed discordant results, where ChatGPT significantly underperformed physicians, achieving only 74% accurate imaging recommendations [26]. Interestingly, the trend in this experiment showed very high sensitivity with low specificity, consistent with ChatGPT's tendency toward caution observed in earlier triage studies [9,10,12,16]. These discrepancies may stem from variations in prompt design, data format, study protocols, and differences in physician practice patterns; however, the level of influence of each of these variables would be impossible to determine presently. Given the current evidence, it is premature to determine whether ChatGPT can reliably guide imaging workup decisions on a broader scale.

Another group studied the use of ChatGPT to interpret radiology reports for clinical urgency. Results were somewhat accurate, with the program identifying urgent radiologic reports with 70% accuracy [27]. However, data on this topic is extremely limited. Additionally, it is common practice at many institutions for radiologists to immediately call ED physicians with urgent results. As most radiology reports are clear enough for a trained physician to decipher quickly, adding ChatGPT into the workflow would most likely be counterproductive. The best use scenario for this technology would be to potentially integrate ChatGPT into the EMR for automated alerts. However, in its current iteration, ChatGPT does not offer a clear advantage for ED clinicians in interpreting urgency or the actionability of radiology reports.

Beyond radiology, ChatGPT has been used to extrapolate various scores from EMR data. The potential in this application would be to have ChatGPT running in the background of an EMR to generate helpful scoring outputs for risk stratification of common conditions. However, unless the scores can be proven consistently reliable, physician oversight would involve manual verification. ChatGPT has been utilized thus far to calculate the following emergency medicine risk stratification scores: the Thrombolysis in Myocardial Infarction (TIMI), HEART, National Institutes of Health (NIH) Stroke Scale, Canadian Syncope Risk Score, Alvarado Score for Appendicitis, and Canadian Head CT [28,29]. Collectively, these studies suggest moderate to high accuracy on a population level, though they also demonstrated significant variability at the individual level [28,29]. Given that manual oversight of these scores still remains essential and ChatGPT offers no benefit in saving time, the immediate utility of ChatGPT in this domain appears limited.

In terms of other miscellaneous decision support applications studied, including admission recommendations, antibiotic prescriptions, and other various ED and ethical scenarios, limited evidence generally shows inferiority to ED residents [26,30]. As noted in multiple prior sections, this inaccuracy tended to arise from its propensity to give overly cautious recommendations. Although data on these specific topics remain limited, the observed patterns suggest that ChatGPT displays a persistently cautious bias. Notable exceptions include antibiotic recommendations, where ChatGPT showed a modest (though not statistically significant) advantage over residents, and ethical problem-solving, in which the model demonstrated unexpectedly strong performance [26-30]. The case of antibiotic recommendations has not been reproduced and may be an artifact of small sample size, but the ethical proficiency is quite interesting. Having scanned large proportions of all human literature for training, ChatGPT is well-versed in diverse schools of ethical and religious thought. Although many fear the ethical implications of AI, ChatGPT's extensive exposure to philosophical texts could give it an advantage in providing ethical guidance beyond what any single individual might offer. This area of research is quite young and cannot be relied upon currently; however, the data demonstrate AI's potential future clinical decision-making value across various domains.

Specialist consultant

A growing body of research now explores ChatGPT's utility as a virtual specialist consultant in the ED. Although the model's performance varies by clinical context, these studies collectively indicate that LLMs may help streamline diagnostic processes, provided that human oversight remains central. Below is a synthesis of the latest findings across multiple specialties, describing both ChatGPT's promise and its current limitations.

Orthopedic Consults 

For orthopedic concerns, Kunze et al. [8] specifically evaluated ChatGPT 4's potential to assist in triaging and managing knee complaints: 20 simulated scenarios (10 for triage and 10 for expanded vignettes) were entered, and ChatGPT 4's differential diagnoses and treatment plans were graded by two orthopedic sports medicine physicians. The top diagnoses were accurate 70% of the time, and 90% of the time, the surgeons' preferred diagnosis appeared in either the first or second position on ChatGPT's list. Diagnostic accuracy reached 100% once the model was provided with more context in the expanded vignettes. Although ChatGPT 4 occasionally provided questionable advice, most of its management recommendations (90%) were considered appropriate. The authors concluded that while ChatGPT 4 can produce clinically reasonable workups and treatments for knee complaints, careful oversight by qualified physicians remains essential. When provided with image data from patient X-rays, Mohammadi et al. [31] documented ChatGPT 4's strong specificity (70%) but weak sensitivity (27.5%) in detecting tibial fractures, whereas an advanced iteration (GPT4o) improved enough to match human experts.

Psychiatric Consults

With the prevalence of psychiatric presentations to the ED, an AI-driven psychiatric consult service would be high-yield to an ED provider. In a small pilot study by Laherrán et al. [32], ChatGPT 4 was given clinical presentations of 15 patients with varying psychological conditions. The model's diagnostic outputs were compared to decisions made by psychiatrists. Although ChatGPT achieved a moderate kappa value of 0.561, the sample size was small, which limited the reliability of conclusions. Nonetheless, these preliminary findings suggest that LLMs may be adept at rapidly narrowing down psychiatric diagnoses.

Infectious Disease Consults

Infectious disease management has likewise been a focus of exploratory research. Maillard et al. [33] tested ChatGPT on a prospective cohort of 44 patients with positive blood cultures, evaluating its performance in diagnosis, workup, empiric antibiotics, and source control. ChatGPT was able to produce the correct diagnosis in 59% of cases, proposed an appropriate diagnostic workup 80% of the time, and recommended proper empiric antibiotics in 64% of cases. It was less successful at selecting definitive antibiotics (36% accurate), and in a small percentage of cases (2-5%), it proposed harmful regimens or inadequate source control. Only 2% of management plans were deemed "optimal." While ChatGPT is not ready to autonomously guide infectious disease consults, this study highlights its potential as a decision support tool, particularly in crafting diagnostic workups that a supervising clinician can then refine.

Gastrointestinal Consults

In evaluating acute ulcerative colitis presentations, Levartovsky et al. [34] studied ChatGPT's performance using the Truelove and Witts criteria to determine disease severity and the need for hospitalization. ChatGPT's recommendations were 80% consistent with gastroenterologists across 20 cases. These findings indicate that ChatGPT could serve as an adjunct to clinical judgment, particularly for time-sensitive decisions like admissions and advanced therapeutics in acute inflammatory bowel disease.

Limitations

Despite its rapid evolution and the continual improvements seen with new models, the use of ChatGPT in the ED is accompanied by notable limitations that must be addressed to ensure safe and effective implementation. While GPT-4.0 demonstrates faster, more precise performance compared to earlier versions, its accuracy still generally falls short of matching a trained physician, and it can occasionally produce incorrect or contradictory information. Further complicating risk, ChatGPT is known to occasionally provide seemingly plausible evidence to support erroneous claims, a phenomenon often referred to as "hallucination." This problem makes it difficult for the end user to discern accurate content from misinformation. Additionally, even though ChatGPT's natural language processing capabilities are robust, the model often struggles to capture the subtleties and atypical presentations that are unique to individual patients [35].

A frequently proposed solution for mitigating errors and building user confidence is for ChatGPT to cite its sources. However, the reliability of these citations is poor: Ghanem et al. found that ChatGPT could only generate accurate sources 43.3% of the time [36]. In the fast-paced environment of an ED, providers depend on accurate, up-to-date references to make life-saving decisions. If clinicians must spend valuable time verifying ChatGPT's citations only to discover irrelevant or unrelated references, the program would be a hindrance rather than a help [37].

Another challenge arises when ChatGPT is exposed to the shorthand and abbreviations routinely found in medical documentation. Two problematic scenarios commonly occur: (1) multiple abbreviations that mean the same thing and (2) identical abbreviations referring to different concepts. For example, "without" is abbreviated in various forms (e.g., wo, w/o, w.o., w-out), which are generally understood in context by human readers but can easily be misinterpreted by ChatGPT. Similarly, abbreviations such as "CP" might refer to "chest pain", "cerebral palsy", or "clinical pathology", while "BS" can mean "bowel sounds", "breath sounds", or "blood sugar". Although ChatGPT's contextual reasoning can clarify some ambiguities, it is far from flawless, and these misunderstandings can introduce confusion or lead to incorrect clinical recommendations.

Ethical and Legal Considerations

In addition to technical limitations, privacy and security concerns pose significant ethical challenges for any AI tool in healthcare. ChatGPT stores users' input on its servers for monitoring and improvement, potentially violating patient privacy if any Health Insurance Portability and Accountability Act (HIPAA)-protected information is shared. Ensuring that no patient-identifying details are included in prompts or that any future AI model is HIPAA-compliant remains a critical prerequisite for widespread adoption in the ED.

Consent and accountability questions also remain unanswered. One of the foundational principles of medicine is informed consent, requiring that patients understand their treatment plans and the rationale behind them. However, AI systems like ChatGPT operate as "black boxes," meaning the underlying logic and decision-making processes are not transparent. Reliance on ChatGPT could be seen as disempowering the patient from understanding medical decision-making. If ChatGPT is integrated more extensively into clinical workflows, clarifying who bears responsibility for errors also becomes crucial: is it the AI's developer, the healthcare provider who relies on the AI, or the institution that implements it? Without clear legal and ethical frameworks, the question of liability remains open and problematic.

Overall, these limitations, including inaccurate or "hallucinated" outputs, unreliable citations, abbreviation misinterpretation, ethical concerns, and privacy issues, underscore the importance of careful integration and ongoing human oversight. Even if this program becomes practically feasible in the applications discussed, these limitations must be carefully weighed when making the decision to implement it in one's own practice.

## Conclusions

The integration of ChatGPT in emergency medicine presents promising opportunities to enhance clinical practice across various specialties, as evidenced by its ability to provide rapid, guideline-aligned suggestions for diagnostics and management. In the high-pressure environment of the ED, this program could potentially serve as a freely available and valuable augmentation tool for clinicians, assisting with provisional insights, narrowing differential diagnoses, and streamlining documentation and communication processes. Areas where ChatGPT could currently be safely integrated into current use are mainly in the synthesis of documents and communication, which is expected for a language model. However, the literature also shows that ChatGPT is borderline at the level of a physician for certain physician-specific tasks and it is rapidly improving between generations. While use for many of these applications still poses more problems than solutions, certain tasks listed above, for example, generating a differential, may be useful as a "rule-in" tool to rapidly generate information that can be edited by the user.

Despite the potential benefits, significant challenges must be addressed before widespread adoption can occur. ChatGPT's reliance on probabilistic language generation raises concerns regarding the reliability of its recommendations, especially in critical scenarios where minor inaccuracies can lead to serious consequences. Therefore, further rigorous, domain-specific research is essential to evaluate its performance in live clinical settings effectively. Ongoing evaluations will be critical as the technology evolves, with future studies focusing on refining its applications and developing guidelines for its safe use. With version 5.0 likely to be released in the near future, it will be interesting to see if performance in ED tasks grows linearly, logarithmically, or exponentially. If growth proves to be exponential, reliable physician decision augmentation in the ED may become a reality in the near future.

## Figures and Tables

**Figure 2:**
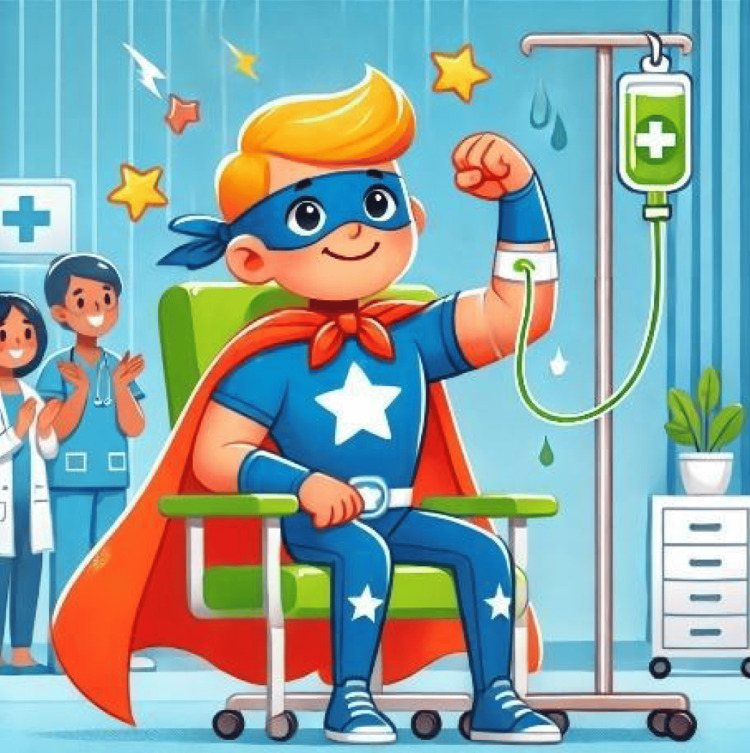
Appendix B, image generation #1

**Figure 3:**
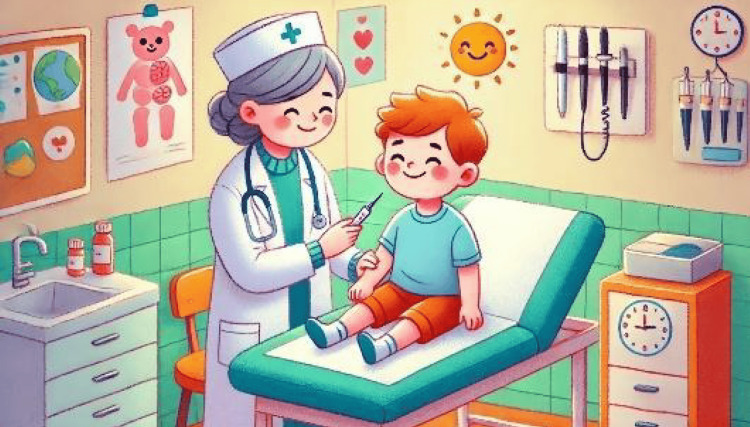
Appendix B, image generation #2

**Figure 4:**
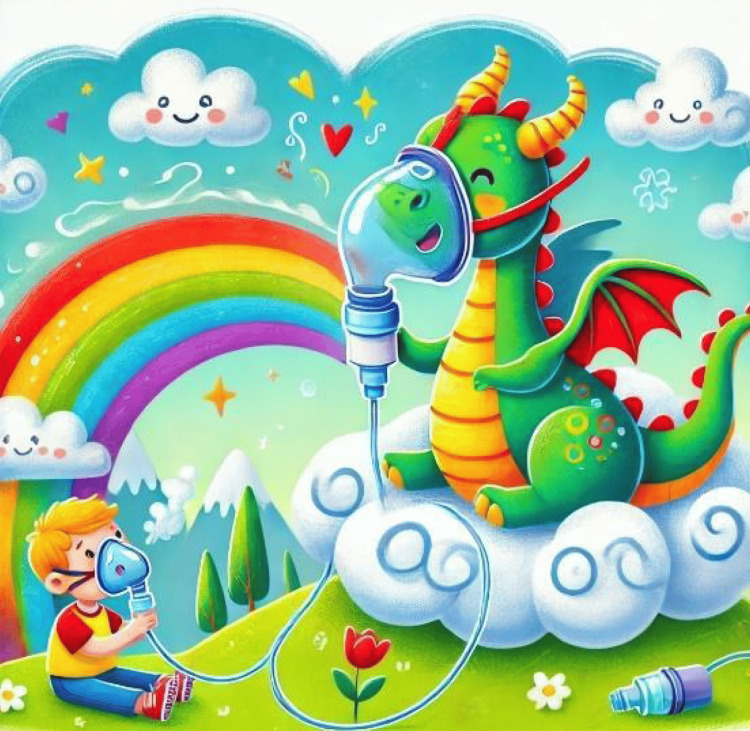
Appendix B, image generation #3

## Appendices

Appendix A

The following, as listed under the section Language Generation Applications, is an example of ChatGPT's note template writing capabilities. The two prompts below were entered into this author's ChatGPT program, which has been trained through regular medical and personal use. The prompts' outputs are without editing and intended to show a real use case for the generation of notes in a physician's own ChatGPT account. The use of these prompts may produce a different response in another user's own ChatGPT account.

"Generate a Procedure Template for Laceration Repair"

Output:

Patient information

● Name:

● DOB:

● MRN:

Pre-procedure evaluation

● Date:

● Time:

● Location:

● Supervising physician (if applicable):

History of injury

● Mechanism of injury:

● Time since injury:

● Location of laceration:

● Contamination present (e.g., dirt, glass, organic material):

● Associated injuries (e.g., tendon, nerve, or vascular involvement):

Patient medical history

● Allergies (e.g., latex, anesthetics):

● Current medications:

● Tetanus immunization status:

● Relevant past medical or surgical history (e.g., diabetes, immunosuppression):

Procedure details

Indication: Repair of laceration to restore the integrity of the skin and prevent infection

Consent

● Informed consent obtained from the patient/guardian, including discussion of risks (infection, scarring, nerve damage) and benefits (wound healing, infection prevention)

Positioning and preparation

● Patient position:

● Area cleaned with (e.g., chlorhexidine, povidone-iodine):

● Draped for sterile field:

Anesthesia

● Type: Local (e.g., 1% lidocaine with or without epinephrine)

● Method: Direct infiltration/nerve block

● Total volume used:

Irrigation and debridement

● Volume and solution for irrigation:

● Debridement performed: Yes/no

Wound examination

● Depth:

● Structures involved: Skin/subcutaneous tissue/muscle/tendon/nerve/vessel

● Foreign body: None/removed

Technique

● Wound edges approximated using:

o Suture material (e.g., 4-0 nylon, absorbable 5-0 chromic):

o Needle type (e.g., cutting, reverse-cutting):

o Pattern (e.g., simple interrupted, running, mattress):

● Staples or adhesive used: Yes/no

Hemostasis achieved

● Method (e.g., pressure, electrocautery):

Post-procedure instructions

● Wound dressing applied: Yes/no (type: e.g., sterile gauze, occlusive)

● Antibiotics prescribed: Yes/no (type and duration)

● Tetanus prophylaxis provided: Yes/no

● Pain management (e.g., acetaminophen, NSAIDs):

● Follow-up for suture removal

o Face: 5-7 days

o Scalp: 7-10 days

o Extremities/trunk: 10-14 days

o Joints: 14 days

Signs to monitor for infection

● Redness, swelling, warmth, increased pain, discharge, fever

Procedure outcome

● No immediate complications observed

● Patient tolerated the procedure well

Physician/provider name: 

Signature:

“Generate a Template for Medical Decision-Making for an Emergency Room Encounter”

Output:

Patient information

● Name:

● DOB:

● MRN:

● Date/time of encounter:

History and presentation

Chief complaint:

History of present illness (HPI)

● Onset:

● Location:

● Duration:

● Characteristics:

● Aggravating/relieving factors:

● Associated symptoms:

● Timing:

● Severity:

Past medical history (PMH):

Past surgical history (PSH):

Medications:

Allergies:

Social history (e.g., tobacco, alcohol, drug use, living situation):

Family history:

Objective findings:

Vital signs

● Temperature:

● Heart rate:

● Blood pressure:

● Respiratory rate:

● SpO2:

Physical exam

● General appearance:

● HEENT:

● Cardiovascular:

● Respiratory:

● Abdomen:

● Extremities:

● Neurological:

● Skin:

● Psychiatric:

Diagnostic studies performed

● Labs (e.g., CBC, BMP, lactate, troponin):

● Imaging (e.g., X-ray, CT, ultrasound, EKG):

● Point-of-care tests:

Medical decision-making

Differential diagnosis

● Primary working diagnosis:

● Other potential diagnoses:

Data reviewed

● Labs/imaging:

● Prior records (if applicable):

● Consultant recommendations (if applicable):

Risk assessment

● Low/moderate/high (e.g., risk of serious morbidity, mortality, or adverse events)

Interventions

● Procedures performed (e.g., laceration repair, joint reduction):

● Medications administered (e.g., analgesics, antibiotics):

● Consultations obtained:

Disposition

● Discharge/home

o Discharge instructions:

o Medications prescribed:

o Follow-up recommendations:

● Admit/observation

o Service:

o Level of care:

● Transfer

o Facility and reason:

● Deceased

o Time of death and actions taken:

Physician's assessment

● Patient's response to evaluation and treatment:

● Justification for the chosen plan of care:

Attending physician

● Name:

● Signature:

Appendix B

The following, as listed under the section Visual Communication and DALL-E Integration, is an example of ChatGPT's image generation capabilities. The three prompts below were entered into this author's ChatGPT program, which has been trained through regular medical and personal use. The prompts' outputs are without editing and intended to show a real use case for the generation of images in a physician's own ChatGPT account. The use of these prompts may produce a different response in another user's own ChatGPT account.

Prompt: Create an image of a cartoon superhero or favorite character "bravely" getting an IV and showing how "strong" it makes them.

In this image, ChatGPT was able to generate a superhero with an IV, exuding a sense of bravery designed to help a child feel more at ease about receiving their own IV.

Prompt: Generate a cartoon image of a brave child getting a lumbar puncture, and make everyone happy to show the procedure is not scary. Disclaimer: This prompt was edited for grammar post-hoc to aid readability. Image generation was not meaningfully affected.

In this image, ChatGPT successfully generated an image that conveyed feelings of warmth and safety; however, it failed to generate an accurate depiction of a lumbar puncture. This difficulty with illustrating less common procedures is a current limitation of ChatGPT's ability.

Prompt: Create an image to depict a nebulizer treatment to educate a child that shows a cartoon dragon blowing smoke/rainbows to show how the mask gives them "magic breath."

In this image, ChatGPT successfully generated a fantastical image of a boy receiving a nebulizer treatment from a dragon. This technology allows doctors to engage pediatric patients by incorporating their interests: in this case, the hypothetical boy's love of fantasy books. By combining this knowledge with a touch of creativity, doctors can create personalized images that make medical treatments feel less intimidating and more enjoyable for young patients.
